# Atomic-level characterization and cilostazol affinity of poly(lactic acid) nanoparticles conjugated with differentially charged hydrophilic molecules

**DOI:** 10.3762/bjnano.9.126

**Published:** 2018-05-02

**Authors:** María Francisca Matus, Martín Ludueña, Cristian Vilos, Iván Palomo, Marcelo M Mariscal

**Affiliations:** 1 Thrombosis Research Center, Department of Clinical Biochemistry and Immunohaematology, Faculty of Health Sciences, Interdisciplinary Excellence Research Program on Healthy Aging (PIEI-ES), Universidad de Talca, Talca, Chile; 2 INFIQC, CONICET, Departamento de Química Teórica y Computacional, Facultad de Ciencias Químicas, Universidad Nacional de Córdoba, XUA5000 Córdoba, Argentina; 3 Laboratory of Nanomedicine and Targeted Delivery, Center for Integrative Medicine and Innovative Science (CIMIS), Faculty of Medicine & Center for Bioinformatics and Integrative Biology (CBIB), Faculty of Life Sciences, Universidad Andres Bello, Santiago, Chile; 4 Center for the Development of Nanoscience and Nanotechnology (CEDENNA), Universidad de Santiago de Chile, Santiago, Chile

**Keywords:** drug delivery, PEGylated nanoparticle, PLA, polymeric nanoparticle, reactive force field

## Abstract

Nanotherapeutics is a promising field for numerous diseases and represents the forefront of modern medicine. In the present work, full atomistic computer simulations were applied to study poly(lactic acid) (PLA) nanoparticles conjugated with polyethylene glycol (PEG). The formation of this complex system was simulated using the reactive polarizable force field (ReaxFF). A full picture of the morphology, charge and functional group distribution is given. We found that all terminal groups (carboxylic acid, methoxy and amino) are randomly distributed at the surface of the nanoparticles. The surface design of NPs requires that the charged groups must surround the surface region for an optimal functionalization/charge distribution, which is a key factor in determining physicochemical interactions with different biological molecules inside the organism. Another important point that was investigated was the encapsulation of drugs in these nanocarriers and the prediction of the polymer–drug interactions, which provided a better insight into structural features that could affect the effectiveness of drug loading. We employed blind docking to predict NP–drug affinity testing on an antiaggregant compound, cilostazol. The results suggest that the combination of molecular dynamics ReaxFF simulations and blind docking techniques can be used as an explorative tool prior to experiments, which is useful for rational design of new drug delivery systems.

## Introduction

In recent years, the use of drug delivery systems based on polymeric nanoparticles (NPs) has generated innovative therapeutic strategies for infection and immune diseases, as well as cancer therapy [1–3]. Polymeric NPs have shown significant advantages compared with many other nanosystems concerning their biodegradation, biocompatibility and highly modifiable physical and mechanical properties [1,4–6]. Polymeric materials also have demonstrated superior efficacy to deliver therapeutic agents at the site of the disease or damaged area and protecting drugs against in vitro and in vivo degradation [7–9].

Poly(lactic acid) (PLA) is one of the most commonly used polymers for the synthesis of NPs. PLA is a synthetic biodegradable, compostable and non-toxic polymer derived from renewable resources [10–12]. Despite its benefits for different formulations in the medical field [13], the applications of PLA are limited, mainly due to its weak hydrophilicity and low drug loading of polar drugs [14]. However, PLA nanoparticles conjugated with hydrophilic molecules like poly(ethylene glycol) (PEG) are a singular kind of functionalized NPs. Several studies have shown that PEGylation of NPs allows improved blood circulation, clearance, biocompatibility and less cytotoxicity [15–19]. Hydrophilic polymer chains at the surface of NPs act as a steric barrier, reducing the opsonization and the subsequent phagocytosis [20–22]. This amphiphilic nature of the copolymer PLA–PEG supports its high potential for development in drug delivery [11] because the PLA core acts as a reservoir for hydrophobic drugs while the corona enables a good dispersion in the blood and offers protection against biological attack [23–24].

In addition to the hydrophilicity/hydrophobicity ratio, the size and surface chemistry are crucial factors in NP–cell interactions. Several studies on metallic NPs, carbon nanotubes, and dendrimers have shown that toxicity of different NPs is determined by a combination of these factors [25–29]. In this respect, computational modelling provides useful approaches to help elucidate the behavior at the atomic level of various nanosystems, predict their drug affinity, and at the same time, reduce the number of preliminary experimental tests (or to be used as a complementary approach to experimental work). The structural characterization in silico provides a comprehensive understanding of properties that govern the formation mechanism and drug loading of NPs and help create better designs and promising compositions. Although there are different experimental methods for synthesis and preparation of PLA–PEG NPs [8,11], molecular descriptions using computational methodologies still are not totally addressed.

Wang et al. [30] carried out dissipative particle dynamics (DPD) simulations to study the PEG–PLA–PEG/paclitaxel delivery system. The results showed a spherical micelle with the drug inside and polymer chains distributed on the surface of the micelle [30]. In a recent investigation [31], it was found that different computational simulation strategies can successfully predict the experimental drug affinity and drug loading for PLA–PEG NPs.

The aim of this work is to characterize at the atomic level the structural properties of differentially charged polymeric NPs PEGylated with DSPE–PEG_(2000)_ (1,2-distearoyl-*sn*-glycero-3-phosphoethanolamine-*N*-[poly(ethylene glycol)-2000]) carboxylic acid-, methoxy- and amino-terminated NPs prepared by the nanoprecipitation method and to study the NP–drug complexation for a model antiaggregant compound, cilostazol [32–33]. PEG chains typically create an interface between the NP core (PLA) and the hydrophilic environment, where the drug encapsulation is largely dependent on the intrinsic affinity between the drug and the PLA core [31]. All-atom molecular dynamics (MD) simulations were performed using the reactive force field (ReaxFF) to prove that the self-assembly process of PLA–PEG NPs is consistent with the experimental method of synthesis [34], investigate the flexibility of polymer chains, and characterize the ability to protect a potential cargo. Docking calculations were employed in order to determine the spatial distribution of cilostazol in polymeric NPs and to explore its potential use in this kind of drug delivery system.

## Experimental

### Characterization of copolymer structures by all-atom molecular dynamics simulations

#### The poly(lactic acid) core

**Figure 1:**
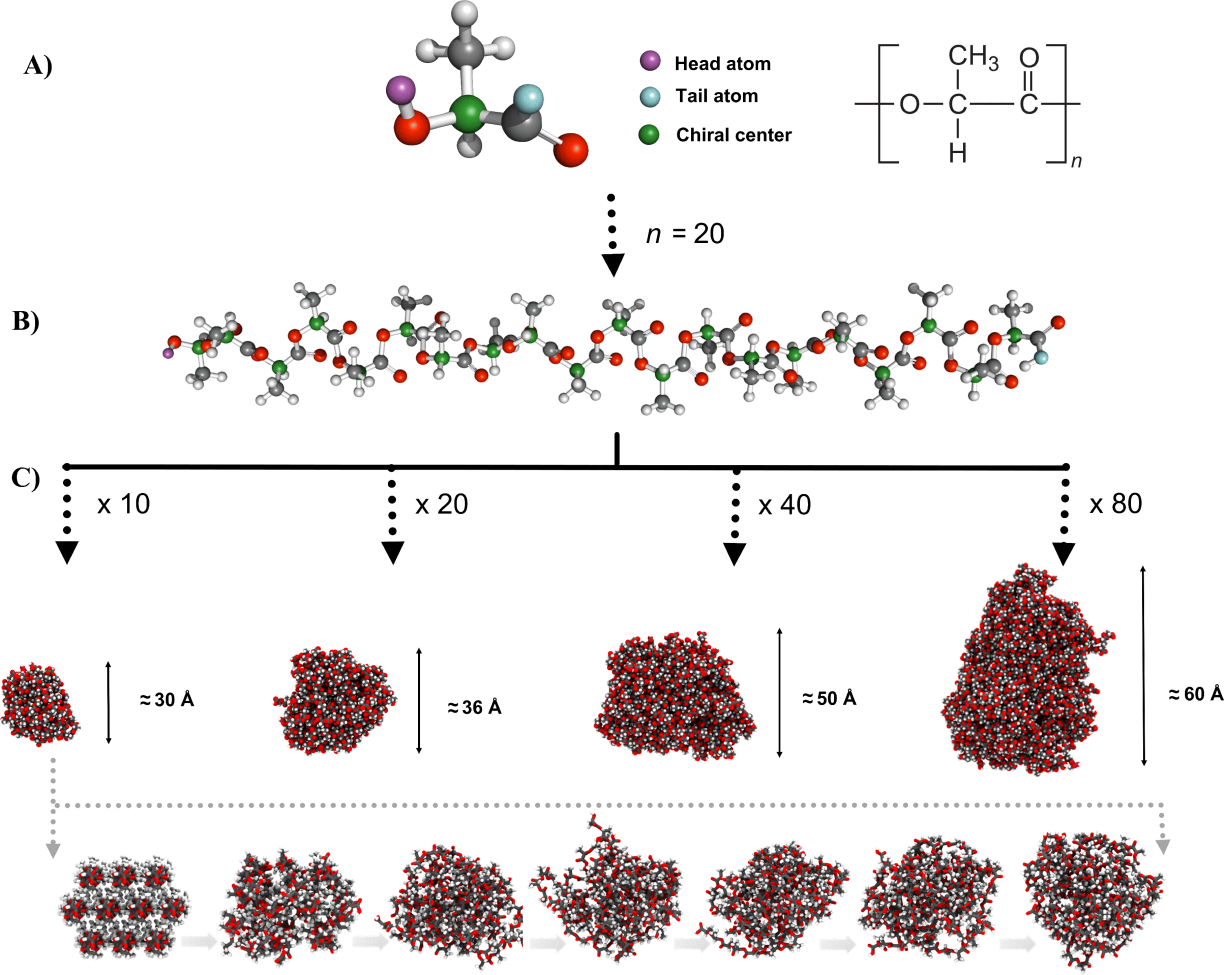
Schematic representation of PLA core formation process. Ball and stick representation of A) lactic acid monomer unit and atom definition for polymeric chain formation, B) poly(D,L-lactic acid) 20 monomer structure and C) van der Waals representation of 10 PLA chains with 20 monomer units after 2 ns of MD simulation and 20 PLA chains, 40 PLA chains, 80 PLA chains with 20 monomer units after 100 ps of simulation. Snapshots in time were taken from the MD simulation of PLA core formation with 10 PLA chains. Atoms are colored according to convention (carbon–gray; hydrogen–white; oxygen–red) except the head, tail and chiral atoms.

PLA polymer chains were built in three-dimensional coordinates with a head-to-tail connection and syndiotactic (D,L) configuration (Figure 1A). The molecular weight distribution of the polymer that was used in the experiments was not well characterized. Thus, considering the limitations on size and computational cost of all-atom MD simulations, the length of the PLA polymer was defined as 20 monomers (Figure 1B).

All MD simulations described in this study were performed using the molecular dynamics simulator, large-scale atomic/molecular massively parallel simulator (LAMMPS) [35–36]. The interactions between atomic species were represented with a novel reactive force field (ReaxFF) [36–37]. This force field considers both covalent and electrostatic interactions by employing a bond-order formalism and a polarizable charge description, enabling depiction of chemical bonding (bond formation and rupture) without expensive quantum mechanics calculations [38–39]. In this work, a modified version of the force field developed by Kim and van Duin, optimized for C/O/H/N/P systems [40], was used.

The convergence criteria for energy minimization, rupture temperature limit and the optimal separation distance for PLA chains to build the PLA core were defined as follows.

**Stopping tolerance for energy:** For a 20-monomer PLA chain, an energy threshold was defined to perform the energy minimization of the system. The stopping tolerance for energy was set as 1.0e^−12^ (means an energy tolerance of one part in 10^12^).

**Rupture temperature limit:** With the aim of creating an initial maximum temperature value (*T*_i_) to be specified at the start of the equilibration phase, a temperature range from 300 to 600 K for a 20-monomer PLA chain was evaluated. The *T*_i_ was set as 600 K.

**Figure 2:**
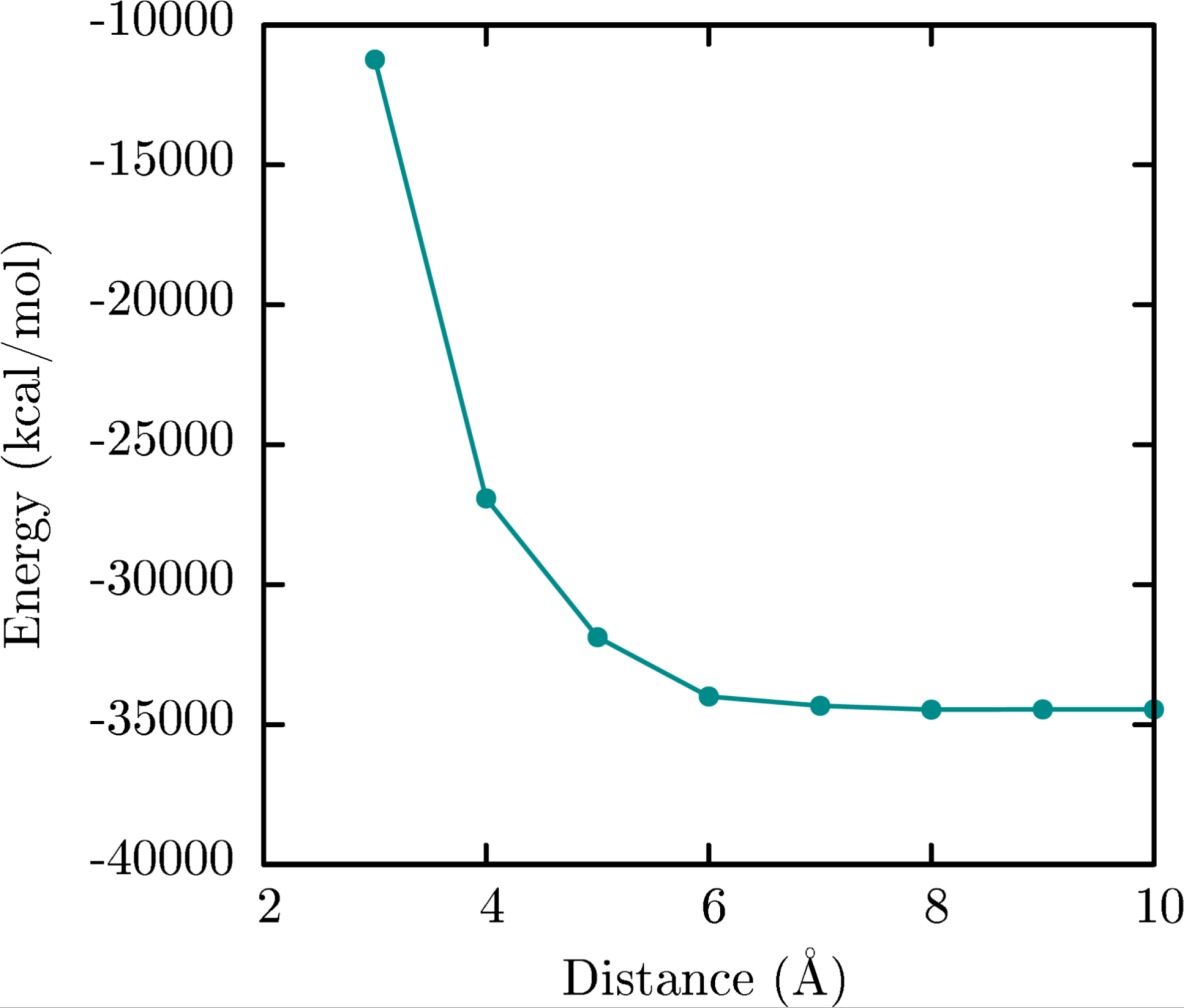
Evaluation of optimal separation distance between PLA chains. The energy was evaluated for two 20-monomer PLA chains at 3, 4, 5, 6, 7, 8, 9 and 10 Å.

**Optimal separation distance:** To ensure an optimal package and minimal energetic repulsion between PLA chains, the optimal separation distance between them was evaluated. The separation distance between PLA chains was established in 7 Å (Figure 2). Thus, 10 PLA chains were packed in a 100 × 100 × 100 Å^3^ periodic box to assemble the PLA core by a MD simulation in vacuum.

The initially packed configuration was minimized using the Polak–Ribiere version of the conjugate gradient (CG) algorithm [41] with a convergence criteria of 1.0e^−12^ followed by an equilibration of 2 ns at 310 K using NVT ensemble (constant temperature and volume) and a timestep of 1 fs. A Nose–Hoover thermostat was used to control the temperature with a damping of 100 timesteps.

To corroborate the correct assembling methodology, several MD simulations of 100 ps were performed for 20, 40 and 80 PLA chains (Figure 1C). Data collected along the trajectories were used to calculate molecular properties such as a radius of gyration and sphericity with the aim of a better characterization of the PLA core shape.

#### DSPE–PEG coating

**Figure 3:**
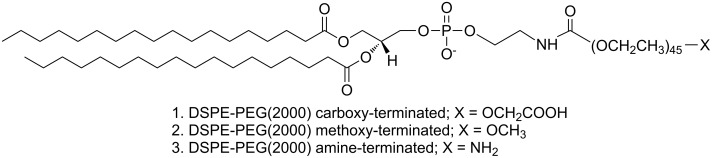
Molecular structure of DSPE–PEG_(2000)_ carboxy-terminated, methoxy-terminated and amino-terminated formulations.

Three PLA formulations were analyzed using DSPE–PEG_(2000)_ carboxylic acid-terminated, methoxy-terminated and amino-terminated molecules (Figure 3) to provide different surface charges, which is a critical factor related to toxicity in nanosystems [25–27,42].

DSPE–PEG structures were built in three-dimensional coordinates. As the starting point, the DSPE–PEG chains were arranged around the PLA core (10 PLA chains) using PACKMOL [43–44], as follows.

- The PLA core was fixed with its center of mass at the center of the system.
- 10 DSPE–PEG configurations were put randomly around the PLA core with the polar head (P atom of phosphate group) of these lipids facing the PLA core while the hydrophilic end (terminal group) of the lipids faced outwards.
- The P atom, representing the polar head, was constrained to be inside a sphere centered at the origin of radius 40 Å, and the atom representing the terminal group was constrained to be outside a sphere of radius 140 Å.

**Figure 4:**
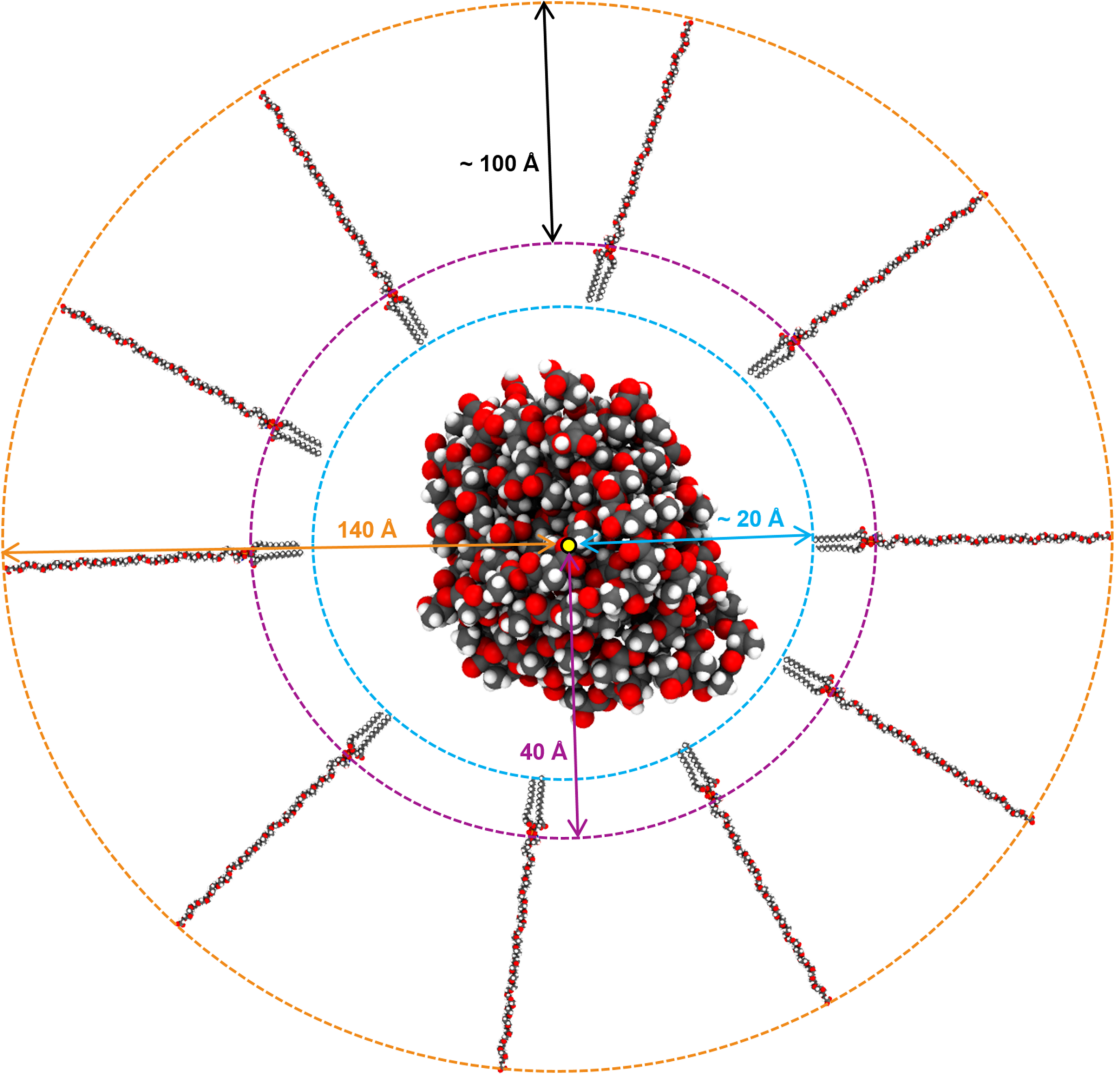
Schematic representation of the initial structure for DSPE–PEG coating process. The PLA core is represented with spheres using conventional color coding. The center of mass is represented by a yellow sphere at the center of the PLA core. The dotted lines represent the different boundary areas, such as core–lipid interface (cyan dotted line), polar zone (purple dotted line) and surface charge (orange dotted line). The distance between the center of mass and the boundary areas is represented using the same color as the dotted lines.

Figure 4 shows the built atomistic model and represents the initial conformation for each case: carboxy-, methoxy- and amino-terminated PLA nanoparticle.

**Figure 5:**
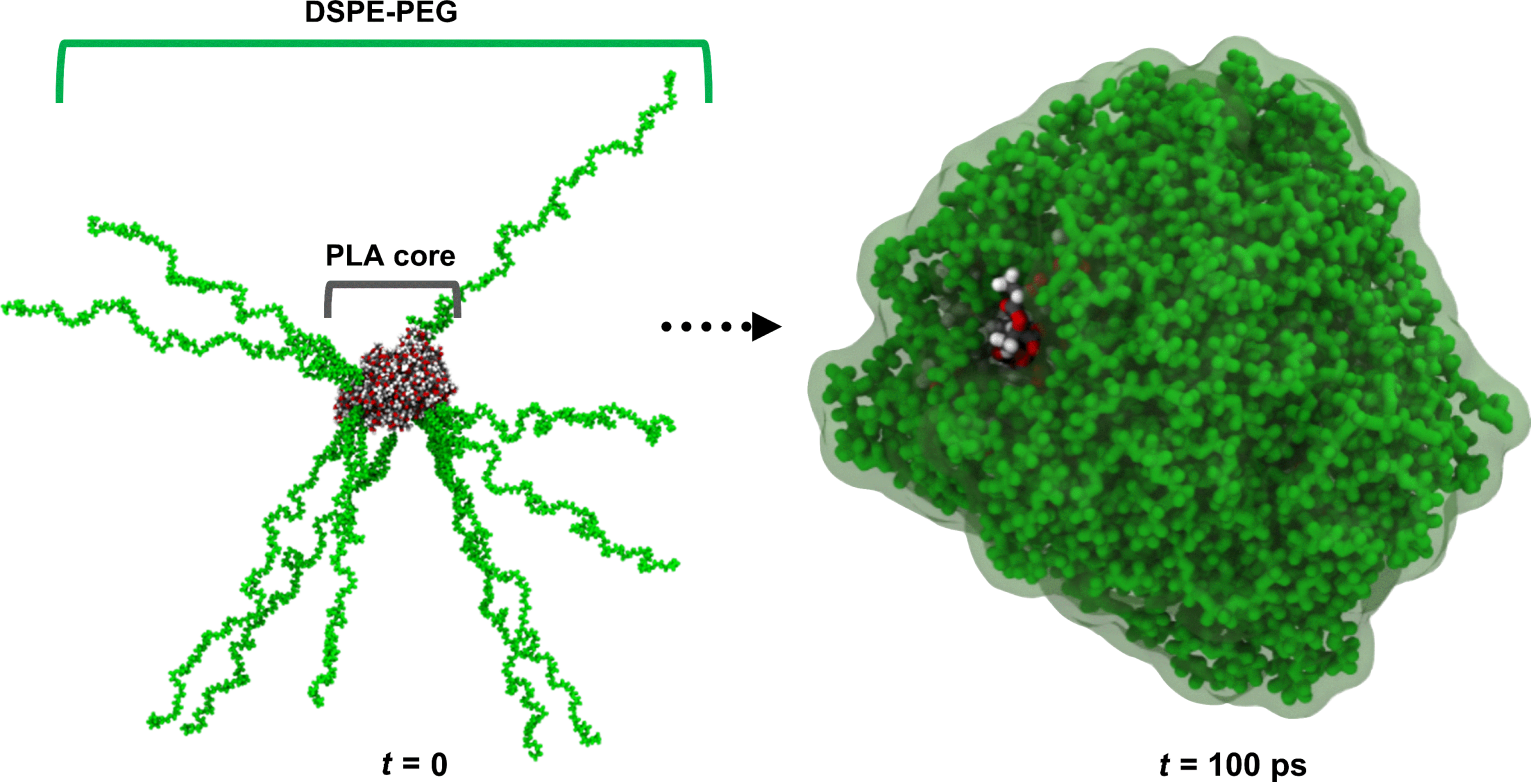
Snapshots of initial (left) and final (right) conformation of PLA–DSPE–PEG-coated structure after 100 ps of simulation. The PLA core is represented with conventional color coding (carbon–gray; hydrogen–white; oxygen–red) and the DSPE–PEG chains are coloured green. Lipid chains fold and cover a significant portion of the PLA core surface.

Then, this starting configuration was also minimized and equilibrated with the same conditions described above, obtaining the final PLA–DSPE–PEG-coated structure (Figure 5).

Finally, to analyze the stability of this structure, and at the same time reduce the computational cost, Langevin dynamics were performed on these final structures with a friction coefficient of 30 ps^−1^. In this way, the solvent particles are omitted, and their effects are represented by a combination of random forces and frictional terms. Simulations of 1 ns were carried out. The velocity Verlet-like algorithm was performed at the time of integration. The temperature was controlled at 310 K with a Langevin thermostat [45] and the pressure was controlled at 1 atm using a Berendsen barostat [46]. The radius of gyration, sphericity and molecular distribution of PLA and DSPE–PEG groups were calculated from the trajectories.

#### Molecular docking

**Figure 6:**
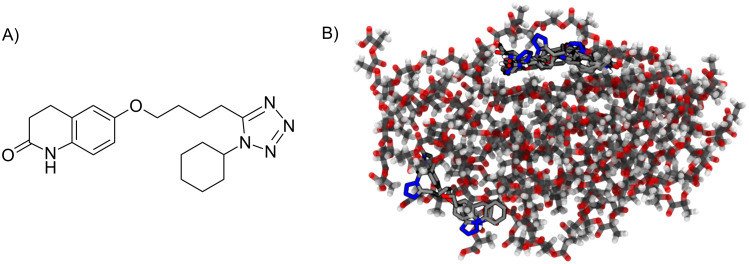
A) 2D structure of cilostazol. B) Main putative binding sites of cilostazol docked into the PLA core. The PLA core is represented with conventional color coding and the chains are displayed slightly transparent. Cilostazol poses are represented in sticks.

Blind docking calculations were performed using AutoDock 4.2 [47] software. The 3D structure of the drug (Figure 6A), was sketched and preoptimized; partial charges were assigned and rotatable bonds were identified. All files for docking calculations were prepared using AutoDock tools (ADT) [48]. The orientation of polymer chains obtained from the above MD simulations was used to carry out the docking calculations. Due to the fact that the polymer core is the protective portion for drugs in this kind of nanocarrier [31], only the PLA core was considered for docking calculations.

AutoDock uses a rapid grid-based method for energy evaluation. A grid volume large enough to cover the entire surface of the PLA core was built (126 × 126 × 126 Å^3^) using a grid spacing of 0.5 Å. The grid parameters were generated using AutoGrid 4.2.6 and the Lamarckian genetic algorithm (LGA) was used to perform a search of the conformational space of the drug. The docking runs were set to 100.

The docking poses were analyzed by examining their binding energy score and the most visited “hot spots” (putative binding sites). The most energetically favorable conformations were selected as the best poses.

## Results and Discussion

### Poly(lactic acid) core

#### Radius of gyration

**Figure 7:**
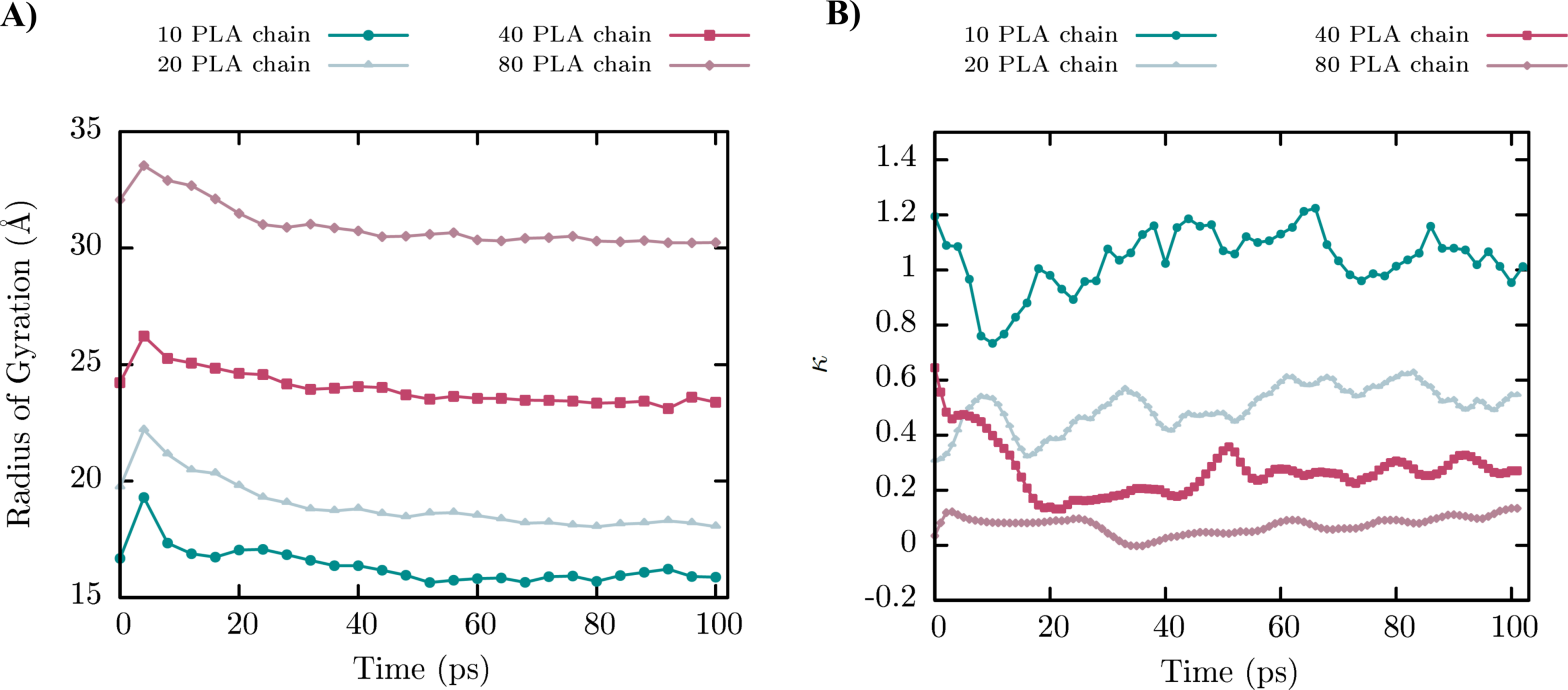
A) The radius of gyration and B) triaxial parameter as a function of simulated time for PLA cores with a different number of PLA chains (10, 20, 40 and 80).

The radius of gyration (*R*_g_) is related to the mean distance of atoms in a molecular structure with respect to its center of mass [49]. The radius of gyration is a good indicator of the spatial conformation of the molecule. Thus, a higher value represents a higher spatial disposition. The radius of gyration was calculated for all PLA core models as a function of simulated time, as shown in Figure 7A.

The mean *R*_g_ were calculated, including the last 60 ps of simulation, ensuring a steady value for each PLA core conformation. For 10 PLA chains, a value of 15.9 ± 0.2 Å was obtained. Meanwhile, a value of 18.3 ± 0.2 Å, 23.5 ± 0.2 Å and 30.4 ± 0.1 Å was obtained for 20, 40 and 80 PLA chains, respectively.

#### Triaxial parameter

To identify the grade of spherical shape for the PLA cores of NPs, a modified script developed by Paz et al. [50] was readjusted, which classifies the geometrical structures of PLA cores into three groups of spheroids: spheres, oblates and prolates [50]. According to the described procedure by Paz et al. [51] to analyze the geometric deformations over time, it is necessary to define the triaxial parameter:

[1]
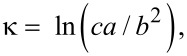


where *a* represents the largest axis of symmetry of the spheroid, *b* is the middle axis and *c* is the smallest axis. Based on this, *κ =* 0 indicates a sphere, *κ >* 0 indicates a prolate and *κ <* 0 denotes an oblate.

Figure 7B shows the evolution of *κ* vs time for the PLA core with a different number of PLA chains. The spherical character increased with a major number of PLA chains. It can be observed that the resulting shape is prolate (*κ >* 0) for 10, 20 and 40 PLA chains.

The sphericity of PLA cores depends directly on the number of chains, independent of their initial configuration. In that context, this behavior is represented by 40 PLA chains (red line) in Figure 7B, in which the initial configuration (at time 0) is more prolate compared with 20 PLA chains (pastel blue line), reaching values of *κ* = 0.64 and *κ* = 0.30, respectively. The initial configuration did not affect the final sphericity of cores because after only a few nanoseconds, both lines intersected. The spherical character of PLA cores reached a steady value for 40 and 80 PLA chains after 60 ps of simulation.

### DSPE–PEG-coated PLA nanoparticles

#### Radius of gyration

**Figure 8:**
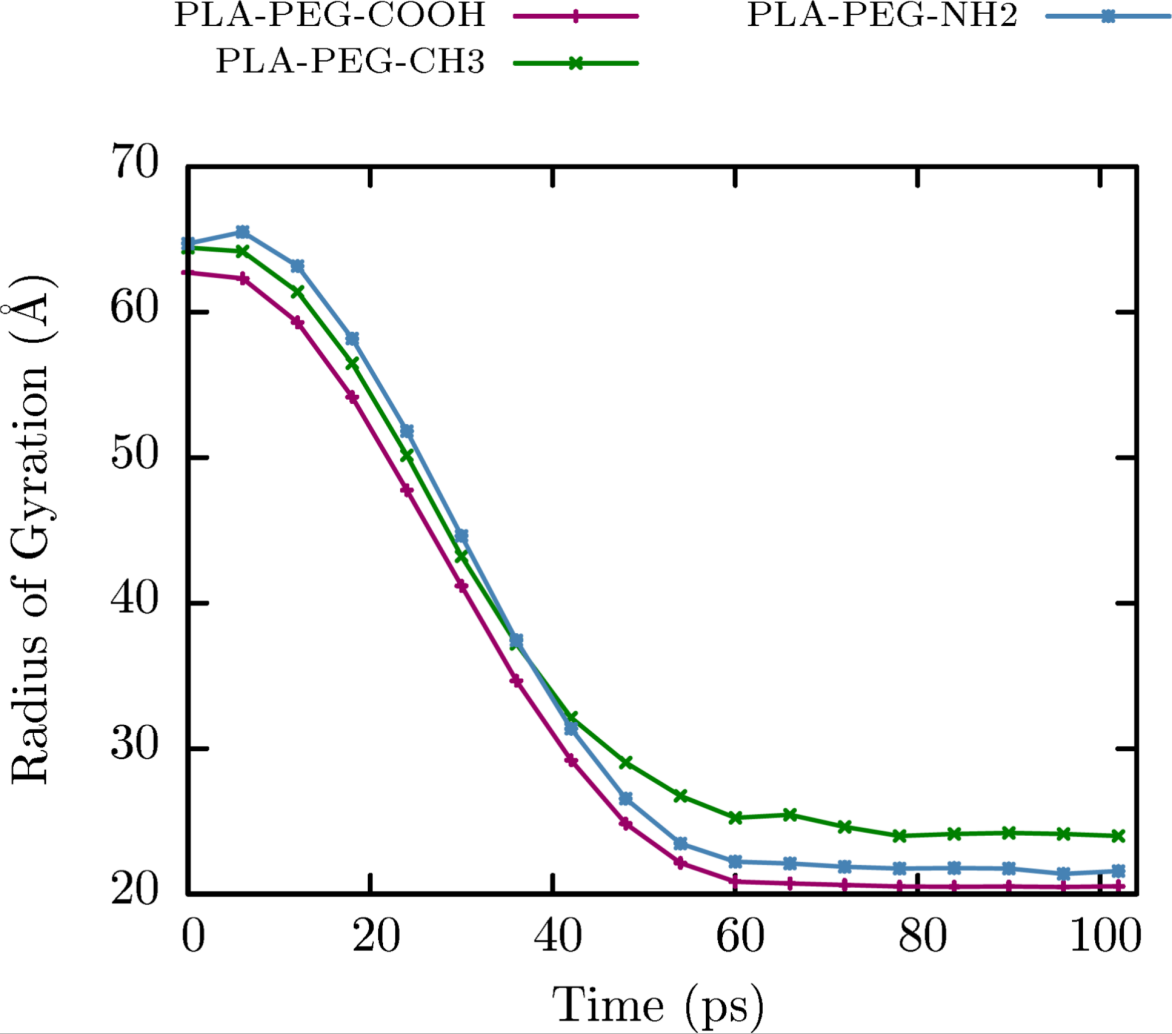
Radius of gyration as a function of time for anionic (PLA–PEG–COOH), neutral (PLA–PEG–CH_3_) and cationic (PLA–PEG–NH_2_) nanoparticles.

The radius of gyration can also characterize the relative DSPE–PEG-coated nanoparticle size and position of the constituent units. The radius of gyration for differentially charged (carboxylic acid-, methoxy- and amino-terminated) DSPE–PEG-coated PLA nanoparticles as a function of time is shown in Figure 8. For carboxylic acid-, methoxy- and amino-terminated molecules, the radius of gyration values were reduced from 62.7 to 20.5, 64.4 to 23.9 and 64.7 to 21.6 Å, respectively.

The different spatial distribution of NPs at the start and at the end of simulation demonstrates the DSPE–PEG-coated process. The initial stretched DSPE–PEG chains were folded around the PLA core, covering the polymer surface (Figure 5).

The mean *R*_g_ values were calculated from the last 40 ps of simulation, where the structure size appeared to stabilize. It was observed that the size of all those DSPE–PEG-coated PLA nanoparticles was homogeneous; however, a slightly larger particle size was found with methoxy-terminated chains (24.5 ± 0.6 Å versus 20.7 ± 0.2 Å for carboxylic acid-terminated and 21.8 ± 0.3 Å for amino-terminated NPs). Interestingly, those results were consistent with experimental results measured by dynamic light scattering [34]. Nevertheless, since an all-atom and reactive force-field model was used, which increased computational cost, a smaller model was built maintaining the conformation of the whole system. This is the reason why discrepancies in scale (≈20 Å and ≈200 nm diameter for computational and experimental results, respectively) were observed.

#### Distribution of DSPE–PEG terminal groups

**Figure 9:**
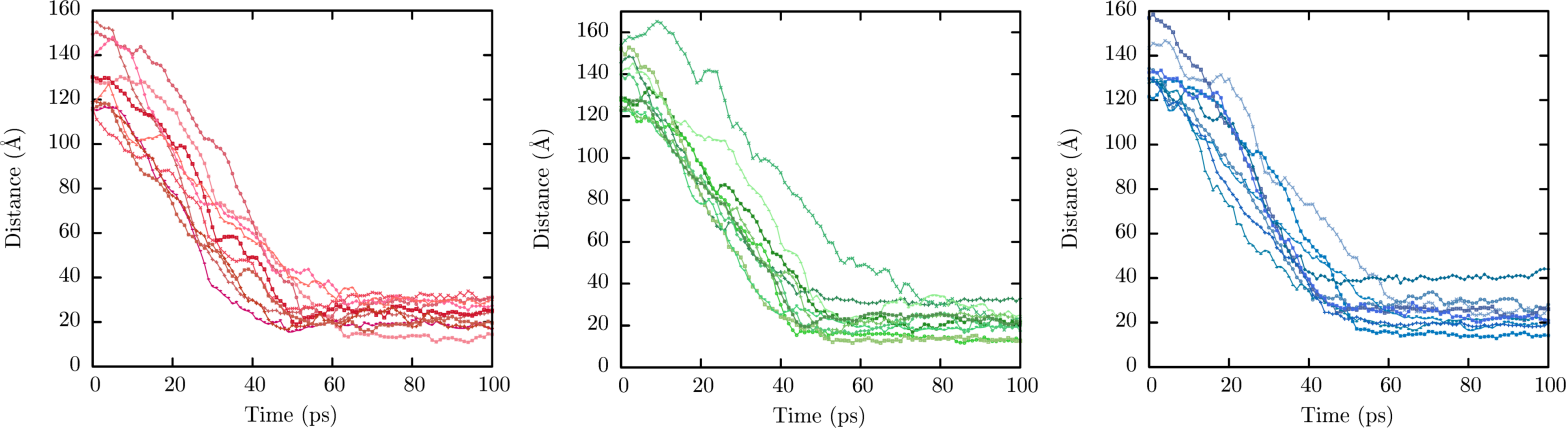
Distance between terminal groups from DSPE–PEG–lipid and center of mass of the PLA core as a function of time. Distances between COOH groups from an anionic nanoparticle and the center of mass of the PLA core are represented in ten different red-styled lines. Distances between CH_3_ groups from a neutral nanoparticle and the center of mass of the PLA core are represented in ten different green-styled lines. Distances between NH_2_ groups from a cationic nanoparticle and the center of mass of the PLA core are represented in ten different blue-styled lines.

To estimate the charge distribution for each NP, the distance between different terminal groups (COOH for anionic NP, CH_3_ for neutral and NH_2_ for cationic NP) and the center of mass of the PLA core was measured. Figure 9 shows the distribution of all terminal groups (ten) of each NP along the simulation.

At the start of the simulation, the terminal groups were observed within 120–160 Å from the center of mass for carboxy-, methoxy- and amino-terminated NPs, which corresponded to the stretched DSPE–PEG chains stage. The distance decreased as simulation time increased in all systems. At the end of the simulations (last 40 ps) the terminal groups were observed at 24.02 ± 5.8, 22.9 ± 6.6 and 24.1 ± 7.1 Å for carboxy-, methoxy- and amino-terminated NPs, respectively. Based on the final values of *R*_g_, it can be suggested that all terminal groups are randomly distributed at the nanoparticle surface. Ensuring a correct PEGylation process is crucial. The surface design of NPs requires that the charged groups must surround the surface region for an optimal functionalization/charge distribution, which is a key factor in determining physicochemical interactions with different biological molecules inside the organism [52].

The interaction energies between the polymeric phase (PLA) and the lipid portion (DSPE–PEG) (Table 1) were similar in all cases and it is governed by hydrophobic interactions. Despite that van der Waals energy was similar in all formulations (−664,536.34, −661,500.71 and −659,315.18 kcal/mol for carboxy-, methoxy- and amino-terminated NPs, respectively), the slightly lower energetic contribution (total energy (*E*_total_), potential energy (PE) and kinetic energy (KD), and van der Waals (vdW) energy (*E*_vdW_)) obtained in the amino-terminated NPs could lead to significant differences in the experimental synthesis, producing particles with a nonuniform size and/or aggregates.

**Table 1:** Interaction energy (kcal/mol) between the PLA core and DSPE–PEG lipid portion.

Nanoparticle	*E*_total_ (PE + KE)	*E*_vdW_	*E*_C_

PLA–PEG–COOH	−685,941.15	−664,536.34	−27,823.22
PLA–PEG–CH_3_	−681,862.84	−661,500.71	−27,321.55
PLA–PEG–NH_2_	−681,010.60	−659,315.18	−27,562.75

#### Characterization of NP–drug complexes

Docking studies showed that cilostazol was trapped in PLA chains and could also be found at the interface of PLA and DSPE–PEG, interacting with both PLA chains and the nonpolar region of the lipids. The high affinity of cilostazol to the PLA core was consistent with the *A* log *P* value (octanol/water partition coefficient) of 3.38 calculated using ALOGPS 2.1 software package [53] and shown in Table 2. Table 2 and Figure 6B show the ten lowest energy conformations of cilostazol and their location within the NP obtained in the docking calculations.

**Table 2:** Ten lowest computed binding energy values for cilostazol. An *A* log *P* value of 3.38 was calculated.

Binding energy (kcal/mol)

−6.90
−6.59
−6.59
−6.53
−6.38
−6.24
−6.17
−6.15
−6.13
−6.09

This NP–drug noncovalent complex was established mainly by hydrophobic interactions, which would allow an easier cargo delivery as the polymer degrades.

It is important to note that this combined computational methodology could be particularly effective in cases where potential drugs have similar hydrophobicity values to achieve a first approximation to guide the experimental phase. It could be expected that a drug with a lower degree of hydrophobicity would be more likely to migrate to the surface of the NP, interacting with the terminal groups of lipid chains (for example through electrostatic interactions) or with the solvent, and thus, the protective function of the nanocarrier would be revoked. However, significant differences in experimental results (drug loading) have been reported when testing drugs with comparable hydrophobicity [54], which could be explained by NP–drug interactions calculated by these predictive tools.

## Conclusion

In the present study, MD simulations provided a comprehensive understanding of the factors that contribute to PLA–PEG nanoparticle formation. To the best of our knowledge, this is the first time that the reactive force field ReaxFF was used to simulate polymer nanoparticle formation.

We have characterized the structure of differentially charged (anionic, neutral and cationic) polymeric DSPE–PEGylated nanoparticles. We detected a self-assembling process in which a core–shell structure is observed with PLA in the core and DSPE–PEG in the shell; this model is consistent with the nanoprecipitation synthesis method previously used [34]. Methoxy-terminated NPs showed a slightly larger size compared to charged particles just as it was observed in the experimental results, and all terminal groups were distributed at the surface of the NPs.

The combination of MD-ReaxFF and blind docking techniques described in this work allowed the investigation of the antiplatelet drug encapsulation ability of polymeric NPs and can be used as a prior explorative tool or as a complementary approach to experiments, which is useful for the rational design of new drug delivery systems.

In future work, this approximation will be correlated with maximum drug loading determined experimentally and the protective role of differentially charged PEG for drug release process will be studied.
